# Comparative Study of the Structure, Properties, and Corrosion Behavior of Sr-Containing Biocoatings on Mg0.8Ca

**DOI:** 10.3390/ma13081942

**Published:** 2020-04-20

**Authors:** Mariya B. Sedelnikova, Yurii P. Sharkeev, Tatiana V. Tolkacheva, Margarita A. Khimich, Olga V. Bakina, Alla N. Fomenko, Aigerim A. Kazakbaeva, Inna V. Fadeeva, Vladimir S. Egorkin, Sergey V. Gnedenkov, Juergen Schmidt, Kateryna Loza, Oleg Prymak, Matthias Epple

**Affiliations:** 1 Laboratory of Physics of Nanostructured Biocomposites, Institute of Strength Physics and Materials Science of SB RAS, Tomsk 634055, Russia; sharkeev@ispms.tsc.ru (Y.P.S.); tolkacheva@ispms.tsc.ru (T.V.T.); khimich@ispms.tsc.ru (M.A.K.); ovbakina@ispms.tsc.ru (O.V.B.); alserova@ispms.tsc.ru (A.N.F.); 2 Research School of High-Energy Physics, National Research Tomsk Polytechnic University, Tomsk 634050, Russia; 3 Faculty of Physics and Engineering, National Research Tomsk State University, Tomsk 634050, Russia; aigerim_@bk.ru; 4 Institute of Metallurgy and Materials Science of A.A. Baikov of RAS, Moscow 119334, Russia; fadeeva_inna@mail.ru; 5 Institute of Chemistry FEB RAS, Vladivostok 690022, Russia; egorkin@ich.dvo.ru (V.S.E.); sls@ich.dvo.ru (S.V.G.); 6 Department of Electrochemistry, Innovent Technology Development, D-07745 Jena, Germany; JS@innovent-jena.de; 7 Inorganic Chemistry and Center for Nanointegration Duisburg-Essen (CeNIDE), University of Duisburg-Essen, 45117 Essen, Germany; kateryna.loza@uni-due.de (K.L.); oleg.prymak@uni-due.de (O.P.); matthias.epple@uni-due.de (M.E.)

**Keywords:** Sr-tricalcium phosphate, Sr-hydroxyapatite, magnesium alloy, micro-arc oxidation, biocoatings, structure, corrosion resistance, cell viability

## Abstract

A comparative analysis of the structure, properties and the corrosion behavior of the micro-arc coatings based on Sr-substituted hydroxyapatite (Sr-HA) and Sr-substituted tricalcium phosphate (Sr-TCP) deposited on Mg0.8Ca alloy substrates was performed. The current density during the formation of the Sr-HA coatings was higher than that for the Sr-TCP coatings. As a result, the Sr-HA coatings were thicker and had a greater surface roughness R_a_ than the Sr-TCP coatings. In addition, pore sizes of the Sr-HA were almost two times larger. The ratio (Ca + Sr + Mg)/P were equal 1.64 and 1.47 for Sr-HA and Sr-TCP coatings, respectively. Thus, it can be assumed that the composition of Sr-HA and Sr-TCP coatings was predominantly presented by (Sr,Mg)-substituted hydroxyapatite and (Sr,Mg)-substituted tricalcium phosphate. However, the average content of Sr was approximately the same for both types of the coatings and was equal to 1.8 at.%. The Sr-HA coatings were less soluble and had higher corrosion resistance than the Sr-TCP coatings. Cytotoxic tests in vitro demonstrated a higher cell viability after cultivation with extracts of the Sr-HA coatings.

## 1. Introduction

Currently, magnesium (Mg) alloys represent materials that are widely used in the industry. These materials are especially required in such areas as automotive industry, aerospace, instrumentation, etc. [1,2]. It should be noted, that in the near future Mg alloys would become one of the most widely used industrial materials due to the combination of their small weight and acceptable strength properties [3,4,5,6]. The density of Mg is 1.7 g/cm^3^ and it is close to the density of human bone. The density of other engineering metals is much higher, for example, the densities of aluminum, titanium, and iron are 2.7, 4.5, and 7.9 g/cm^3^ respectively. Moreover, Mg is one of the most common elements of the Earth’s crust; therefore, it is widely spread, and its alloys can be used in various fields [2]. The advantage of Mg is the simplicity of its processing by various methods, such as high-speed milling, turning, rolling, and extrusion. Due to good casting ability of Mg, it is possible to manufacture complex shape parts using a high-performance method, for example, high pressure die casting [1]. Mg alloys demonstrate very good mechanical properties [3] and are non-toxic to the environment and the human body, therefore, they can be used in creating of biodegradable implants [7].

Metal implants, which are frequently used in traumatology, orthopedics, and maxillofacial surgery, are produced from titanium and stainless steel [8]. Although these materials have high strength, wear resistance, and corrosion resistance, their disadvantage is that they lead to the effect of bone stress shielding, which occurs due to the difference in the elasticity modulus (~105 GPa for titanium and 27 GPa for bone) [8]. This consequently leads to bone degradation and osteoporosis. In addition, when using inert titanium implants, repeated implant removal surgery are often required. This is especially relevant in maxillofacial surgery, since repeated surgery can lead to an increase of scarring and the inability to use aesthetic surgery. Use of biodegradable implants that can be metabolized in human body [7,8,9,10,11,12,13,14,15] could solve previously described problems. Cardiovascular (stents) or temporary orthopedic devices that are used in the body only during the recovery period are the examples of bioresorbable Mg alloy devices [1]. In general, any metal that readily corrodes in aqueous solutions and is considered non-toxic can serve for biodegradable implants [13,14]. However, most of the research works in this area have been performed on Mg alloys. Several elements, such as Al, Zn, Ca, Cu, Mn, Zr, Y, and rare earth elements (REs) are commonly used in Mg alloys [1,2,15,16,17,18,19,20]. Ca, Zr, and Zn are elements that are the most often used in medical alloys. Zn and Cu are demonstrating antibacterial properties [20]. In addition, Zn can also improve the mechanical strength and the corrosion resistance of Mg-based alloys [4,16]. In a number of works [4,17,18,19] binary Mg–Ca alloy for orthopedic applications was developed. Although almost total dissolution of Mg–Ca alloys occurred after 3 months, their good tolerance and biomechanical properties were observed. In this case, no systematic toxicity was detected. Osteoblasts demonstrated high activity around the implant [17,18,19].

A huge problem limiting the application of Mg alloys as materials for medical implants is their rapid bioresorption in human body. Mg has the most negative electrochemical potential in comparison with all structural metals and will be polarized anodically in the process of galvanic interaction with other components [1]. In this case, the process of hydrogen evolution, which occurs on anodically polarized Mg surfaces, is a key feature of the electrochemistry of Mg and its alloys [21]. Materials based on Mg are easily oxidized in the presence of water due to the low thermodynamic stability, resulting in a release of H_2_ gas from the water [20]. Moreover, the formation of a protective barrier MgO/Mg(OH)_2_ layer can occur only in an alkaline environment without chloride ions which are present in body fluids [1]. The quantity of H_2_ gas released depends on the speed of magnesium material bioresorption. If the emission of hydrogen gas is rather slow, it can be transported from the initiation place, thus eliminating local accumulation of significant volumes of gas [21].

Surface modification of a magnesium alloy with a protective anti-corrosion coating is an excellent solution to this problem. In addition, the coating can not only slow down the rate of dissolution, but also enhance the processes of implant osteointegration and new bone formation [22,23,24,25]. There are many ways to form biocoatings, for instance an immersion method [22], hydrothermal synthesis [23,24,25], biomimetic [26], sol-gel [27,28], plasma deposition [29,30], high-frequency magnetron sputtering [31,32], micro-arc oxidation (MAO) [3,8,33,34], spraying [3] and high-velocity oxy-fuel spraying [35]. The most popular method of the magnesium alloys surface modification is plasma electrolyte oxidation (PEO) also known as micro-arc oxidation (MAO), microplasma oxidation or anodic spark deposition [36]. Plasma discharges migrate randomly over the surface of the processed material during this treatment, resulting in the formation of thick porous coating [36,37,38,39].

The rate of coatings dissolution and their biological properties depend on the substances that the coatings are consisted of. The most common biocoatings are based on the calcium orthophosphates such as hydroxyapatite (HA) [22,25,27,40] and tricalcium phosphate (TCP) [34] or include their combinations. Hydroxyapatite has the lowest dissolution rate in body fluids. Acid calcium orthophosphates such as tricalcium phosphate [34], brushite [41], and monetite [42] dissolve faster [43]. Thus, by combining various phases, it is possible to control the dissolution rate of biocoatings and thereby to manage the bioresorption of magnesium alloys. Some studies are devoted to the development of silicate coatings on magnesium alloy. Rau et al. [19] used the glass–ceramic coatings on Mg–Ca alloys to reduce its biodegradation rate under physiological conditions. In addition, Dou et al. [44] demonstrated the influence of the second-step voltages on the structural and properties of the coatings on Mg–Zn–Ca alloy.

An important aspect of the effect of coatings on the rate of magnesium alloys dissolution is the phenomena of the simultaneous enhancement of bone regeneration processes. It is known that strontium (Sr) enhances the processes of pre-osteoblastic cells replication and bone formation [45,46,47,48,49,50,51,52,53,54,55,56,57]. Many studies are related to the production of Sr-incorporated coatings on the surface of bioinert titanium alloys [45,46,47,48,49,50,51,52]. However, the works devoted to the surface modification of bioresorbable magnesium alloys by Sr-containing coatings [53,54,55,56,57] are the most interesting to consider.

Sr is a natural bone element, which is generally localized in bone tissues. Chemical and physical properties of Sr are similar to Ca. When Sr^2+^ partially replaces Ca^2+^ in hydroxyapatite structure, the bone formation increases which also leads to an increase in bone mass and bone strength [53,54]. Therefore, medicaments containing Sr are used in the treatment of osteoporosis. The local release of Sr^2+^ promotes osteoblastic response, so Sr-containing calcium phosphate coating at the interface between the implant and the bone stimulates new bone formation [55,56,57]. Analysis of the available researches shows that, effect of phase composition and dissolution rate of the Sr-containing coatings on the amount of Sr released by them and their biological properties require further studies.

The target of the present work was a comparative study of the formation of micro-arc coatings with the participation of Sr-substituted hydroxyapatite (Sr-HA) and Sr-substituted tricalcium phosphate (Sr-TCP), as well as the study of their phase and elemental composition, physicochemical and electrochemical properties, behavior in biological fluid, and toxicity with respect to cell test cultures.

## 2. Materials and Methods

### 2.1. Sample Preparation

Magnesium alloy Mg–0.8 wt.% Ca (Mg0.8Ca) used as substrate material was developed at Helmholtz Zentrum (Geesthacht, Germany). The alloy was produced in accordance with the technology presented in previous studies [34]. Experimental samples 10 × 10 × 1 mm^3^ from Mg0.8Ca were polished by silicon carbide sandpaper No. 1200 grit. Then machined samples were cleaned ultrasonically in distilled water within 10 min. As a result, the roughness (*Ra*) of samples reached 0.3–0.6 μm. Micro–Arc 3.0 installation was used to realize the MAO method for deposition of coatings as reported in the previous papers [33,34]. The electrolyte solution consisted of the following components: Na_2_HPO_4_, 10–30 g/L; NaOH, 3–5 g/L; NaF, 1.5–3.0 g/L; and Sr-HA (Ca_7.5_Sr_2.5_(PO_4_)_6_(OH)_2_) or Sr-TCP (Ca_2_Sr(PO_4_)_2_), 40–60 g/L. Powders of Sr-HA (University of Latvia, Riga, Latvia) and Sr-TCP (Institute of Metallurgy and Materials Science of A.A. Baikov of Russian Academy of Sciences, Moscow, Russia) were obtained by chemical precipitation method. The analysis by X–ray powder diffraction method confirmed the phase composition of the powders. Particle sizes varied within of 1–5 μm.

The MAO process was carried out using the anodic potentiostatic regime with the following parameters obtained during previous research [34,57]: pulse frequency of 50 Hz, pulse duration of 100 μs and the process duration of 5 min. The pulsed voltage varied in the range of 350–500 V.

### 2.2. Experimental Methods

Coatings were deposited on Mg0.8Ca alloy. Their microstructure, morphology and elemental composition were studied with Quanta 400 scanning electron microscope (SEM, FEI Company, Hillsboro, OR, USA) with Genesis 4000 energy-dispersive X-ray analysis installation in University of Duisburg-Essen (EDAX, AMETEK, Mahwah, NJ, USA) and with JEM-2100 transmission electron microscope (TEM, Jeol Ltd., Musashino, Akishima, Tokyo, Japan) in “Nanotech” Common Use Center of Institute of Strength Physics and Materials Science Siberian Branch of Russian Academy of Sciences (Tomsk, Russia). In accordance with ASTM E1382–9 and DD ENV 1071-5, the coatings average pore sizes were measured with help of the secant method. Quantity of secant for each specimen was 50. Average roughness (*R_a_*) was estimated as characteristic of surface roughness. Estimation was carried out with a Hommel–Etamic T1000 profilometer (Jenoptic, Jena, Germany) in National Research Tomsk Polytechnical University. The measured profile characteristics were following. Traverse length was 6 mm and measuring rate was 0.5 mm/s.

X-ray powder diffraction (XRD) method allowed determining the coatings phase composition. XRD was performed with Bruker D8 ADVANCE diffractometer (Bruker, Billerica, MA, USA) in Cu Kα radiation in the angular range from 5° to 90° 2𝜃 in University of Duisburg-Essen (Germany). Scanning step was 0.02°. XRD-profiles were interpreted and phases were identified with Joint Committee on Powder Diffraction Standards (JCPDS) powder database. The volume ratio of crystalline and amorphous phases was calculated from the XRD profiles by quantitative Rietveld refinement [58,59].

### 2.3. Electrochemical Studies

Electrochemical properties of the coated samples and bare alloy were studied with the help of VMC-4 (PAR, USA). The experiments were taken in a three-electrode cell in 0.9% NaCl. A platinum coated niobium mesh and Ag/AgCl electrode were used as a counter and reference electrode, respectively. The working electrode area equaled 0.5 cm^2^.

Linear polarization resistance experiment was performed at a scan rate of 0.167 mV/s in the range ±30 mV vs OCP. The polarization resistance, *R*_p_, was calculated as recommended in ASTM G59-97 [60]. Potentiodynamic polarization (PDP) curves were acquired at 1 mV/s [61]. The values of corrosion potential, *E*_C_ and corrosion current density, *j*_C_ were calculated using [62]:

(1) $$j=j_{C}(10^{(E-E_{C})/\beta a}+10^{-(E-E_{C})/\beta_{c}})$$

EIS—electrochemical impedance spectroscopy measurements, were performed using a 10 mV r.m.s. sinusoidal perturbation signal (0.1 MHz–0.01 Hz; logarithmic sweep 10 points/decade) at an open circuit potential. Versa STUDIO (Princeton Applied Research), ZView and CorrView software (CorrWare/CorrView Software, Scribner Associates, AMETEK, Mahwah, NJ, USA) were used for the experiments control and analysis. Each experiment was performed on three different samples.

### 2.4. Biological Studies

Standard 0.9% NaCl solution was used as environment for the samples biodegradation study according to ISO 10993-5. Both bare Mg alloy samples and coated samples were immersed in the solution at 37 °C during 11 days.

Loss of the sample mass was calculated by the formula:

(m_0_ − m_i_/m_0_) × 100% (2)

where m_0_—mass before the dissolution and m_i_—mass after the dissolution.

L-929 mouse fibroblasts cells line were obtained from State Research Center of Virology and Biotechnology, Vector (Koltsovo, Russia).

The cells were grown as a monolayer in MEM with addition of 10% fetal bovine serum (HyClone, Marlborough, MA, USA), 2 mM L-glutamine (HyClone, Marlborough, MA, USA) and 1% penicillin/streptomycin (HyClone, Marlborough, MA, USA). The cell cultivation was performed at 37 °C and 5% CO_2_ within 24 h. The final cell concentration was 1 × 10^4^ cells in 100 μL per well of a 96-well microplate (TPP, Trasadingen, Switzerland).

The samples were extracted for 3 h at a surface/volume ratio equal to 1 cm^2^/mL of cell medium. A sample of the medium was incubated under similar conditions and was used as a negative control. The obtained extracts were used MTT assay based on 3-(4,5-dimethylthiazol-2-yl)-2,5-diphenyl tetrazolium bromide, which is based on the reaction of MTT reagent reduction with reductases of living cells to formazan stained in purple. The incubation with MTT solution was carried out for 2 h at 37 °C and 5% CO_2_. The optical density was determined on a microplate spectrophotometer Thermo Scientific Multiskan FC (Thermo Fisher Scientific, Waltham, MA, USA) at a wavelength of 570 nm. Parametric methods with a confidence level of p ≤ 0.05 were used for statistical data processing. The determination of the pH of the cell medium was performed during the preparation of the extract within 24-h period.

## 3. Results

### 3.1. Formation, Structure and Morphology of the Coatings on Mg0.8Ca

**Figure 1:**
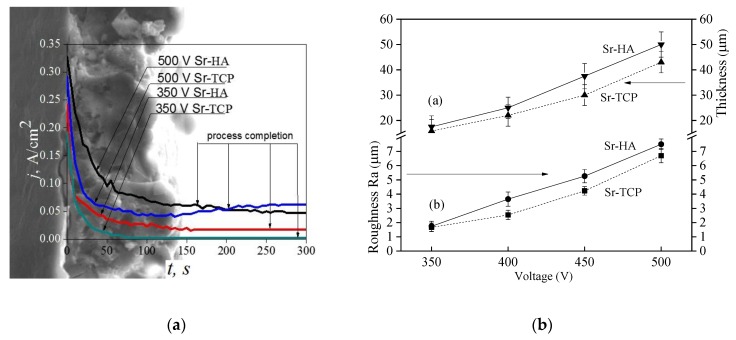
The graphs of the current density versus the process duration for deposition of the Sr-substituted hydroxyapatite (Sr-HA) and Sr-substituted tricalcium phosphate (Sr-TCP) coatings on Mg0.8Ca (**a**) and the graphs of the coatings thickness and roughness Ra versus the applied voltage (**b**).

An anode potentiostatic mode with fixed voltage was used for the Sr-HA and Sr-TCP coatings deposition. The formation and growth of the dielectric coating occurred during the MAO process, while the current density decreased due to the rise in coating thickness (Figure 1a). Higher current densities were observed for Sr-HA deposition process. For example, the initial current density for Sr-HA coating at the applied voltage of 500 V was 0.35 A/cm^2^. In this case, the initial current density for Sr-TCP coating was 0.30 A/cm^2^. For both types of coatings, the current density decreased to a minimum constant value during 1 min of the process deposition.

The coatings had a gradient porous structure (Figure 1a) due to high current density in the initial period of the process formation. The inner layer of the coatings was enriched with pores while the outer layer had a denser structure. Such a gradient porous structure is common for the coatings deposited in the unipolar current mode [63].

The dependencies of the coatings thickness and roughness on the applied voltage are shown in Figure 1b. The graphs show that with the voltage increasing the coatings thickness and roughness also increase. It should be noted that the values of thickness and roughness for Sr-HA coatings were slightly higher than those for Sr-TCP coatings. The maximum thickness and roughness were 50 and 7 μm, respectively for Sr-HA coatings and 40 and 6 μm, respectively for Sr-TCP coatings. This is due to higher current densities, and, consequently, to more intense micro-arc discharges arising during the deposition of Sr-HA coatings.

**Figure 2:**
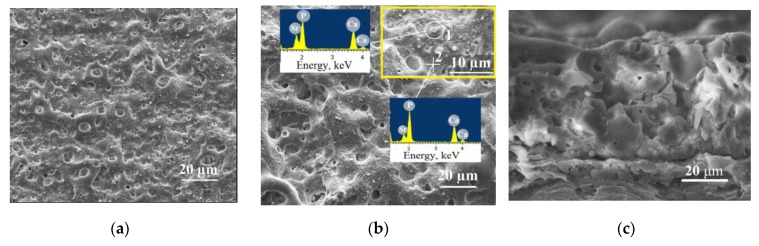

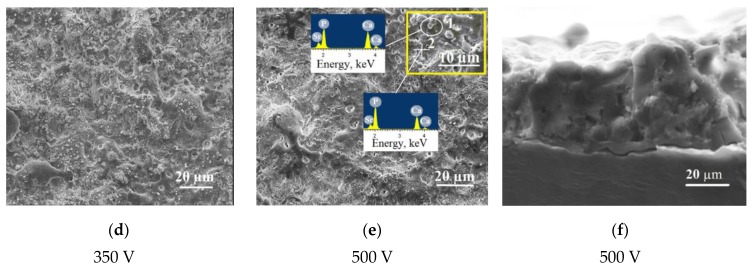
Scanning electron microscope (SEM) images of the surface (**a**,**b**,**d**,**e**) and cross-section (**c**,**f**) of the Sr-HA (**a**–**c**) and Sr-TCP (**d**–**f**) deposited at 350 V (**a**,**d**) and 500 V (**b**,**c**,**e**,**f**). SEM image of the coating surface with higher magnification is presented in the yellow frame.

**Figure 3:**
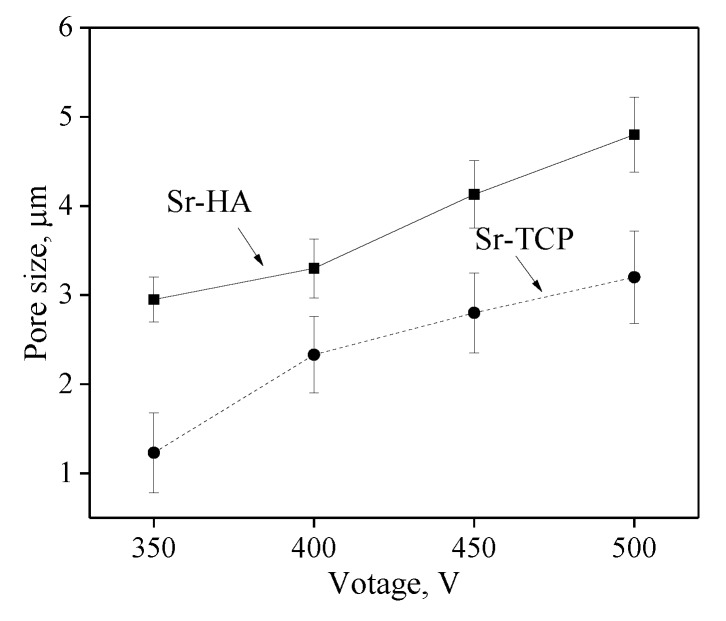
Graphs of the pore sizes against the applied voltage for Sr-HA and Sr-TCP coatings.

The SEM-images (Figure 2) demonstrated the rough relief and the presence of a large amount of pores on the coatings surface. It should be noted that the pore sizes in Sr-HA coatings were almost two-times larger than those for Sr-TCP coatings (Figure 3). The formation of pores occurred due to the action of strong discharges. Thus, the MAO process was more intensive during the formation of Sr-HA coatings. In addition, the particles with sizes of 1–3 µm were distributed uniformly over the surface of the coatings as shown in the micrographs.

Elemental energy-dispersive X-ray analysis (EDX) showed a high content of Ca and P in these particles (Figure 2b,e). For the Sr-HA and Sr-TCP coatings the (Ca + Sr)/P ratio was 0.9 ± 0.1 and 1.04 ± 0.05, respectively in the particles (zone 1). While in the coating regions without particles (zone 2), the ratio (Ca + Sr)/P was lower and equal to 0.7 ± 0.08 and 0.82 ± 0.05 for the Sr-HA and Sr-TCP coatings, respectively. Obviously, these are particles of Sr-containing calcium phosphates that have been deposited on the surface of the coating at the completion stage of the MAO process (Figure 1a). The analysis of SEM images of the coatings cross-section allows us to conclude that there are no particles in the inner layers of these coatings (Figure 2c,d).

In the previous studies, we also observed the particles of β-TCP diffusing from the electrolyte to the coatings surface [34,57].

### 3.2. Structured–Phase and Elemental Compositions of the Coatings on the Mg0.8Ca

**Figure 4:**
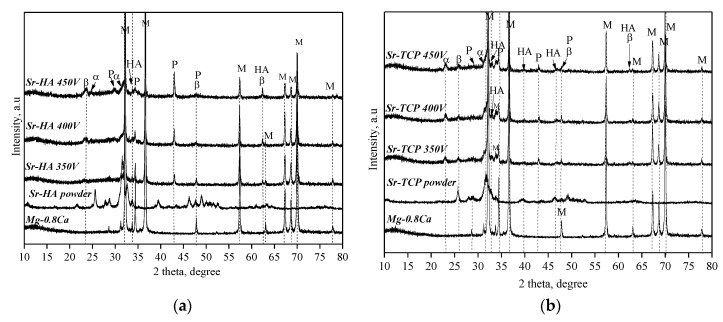
X-ray powder diffraction (XRD) patterns of the Sr-HA (**a**) and Sr-TCP, (**b**) coatings deposited on Mg0.8Ca under different voltages; β—β-Ca_3_(PO_4_)_2_, α—α-Ca_3_(PO_4_)_2_, HA—Ca_10_(PO_4_)_6_(OH)_2_, P—Mg_3_(PO_4_)_2_, and M—magnesium.

**Figure 5:**
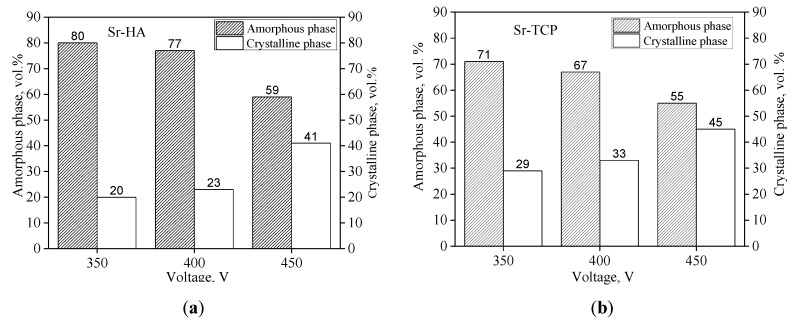
Diagrams of the ratio of amorphous and crystalline phases in the Sr-HA (**a**) and Sr-TCP (**b**) coatings.

Crystalline phases of β-Ca_3_(PO_4_)_2_ (β-TCP), α-Ca_3_(PO_4_)_2_ (α-TCP), Mg_3_(PO_4_)_2_, and Ca_10_(PO_4_)_6_(OH)_2_ hydroxyapatite (HA) were revealed in the coatings of both types with the help of X-ray powder diffraction (XRD) (Figure 4). In addition, reflexes corresponding to the substrate material Mg were presented in the XRD patterns. However, with increasing of the applied voltage the intensity of the reflexes corresponding to the magnesium substrate decreased due to higher thickness of the coatings. XRD analysis showed the presence of an amorphous and a crystalline phase in the coatings (Figure 5). The high temperature generated by strong discharges led to plasma formation and plasma chemical reactions. The reaction products precipitated at the walls of the micro-arc discharges channels and sealed them [36,63]. The amorphous and crystalline phases were formed in the products of plasma chemical reactions due to their rapid cooling and solidification.

The diagrams presented in Figure 5 show the change in the ratio of amorphous and crystalline phases depending on the applied voltage. It should be noted that quantitative ratio of amorphous and crystalline phases was determined with the Rietveld refinement method [58,59]. All of the background areas of diffuse scattering on X-ray profiles were considered as those from the amorphous phase and all the peaks were considered as those from the crystalline phases. However, we excluded all reflexes of substrate (peaks of Mg phase) from the calculation as we aimed to determine the ratio of amorphous and crystalline phases in the coatings not considering the substrate. As can be seen, in the voltage range of 350–450 V the amount of the amorphous phase decreased from 80 to 59 vol.% and from 71 to 55 vol.% for the Sr-HA and Sr-TCP, respectively, and more crystalline phase was formed in the coatings. The formation of a larger quantity of the amorphous phase in the Sr-HA coatings was an extra piece of evidence of the more intensive micro-arc discharges that occurred during the deposition of these coatings.

**Figure 6:**
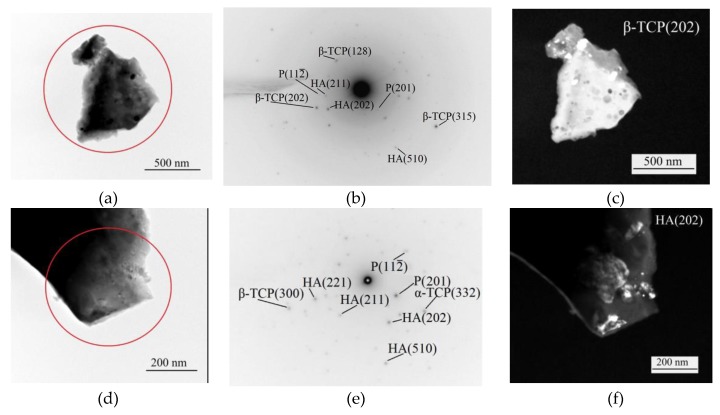
The bright-field (**a**,**d**), dark-field (**c**,**f**) transmission electron microscope (TEM) images and selected area diffraction (SAD) pattern (**b**,**e**) of the Sr-HA (**a**–**c**) and Sr-TCP (**d**–**f**) coatings deposited at 400 V.

The phase composition was confirmed by the TEM results (Figure 6). The pinpoint reflexes were observed in the selected area diffraction (SAD) patterns obtained from the fragments of coating particles (Figure 6b,e), which indicated the presence of crystalline phases in the coatings. The SAD pattern indication showed the presence of the following phases: β-TCP with rhombohedral lattice (JCPDS No. 09-0169), α-TCP with monoclinic lattice (JCPDS No. 09-0348), HA with hexagonal lattice (JCPDS No. 09-0432), and Mg_3_(PO_4_)_2_ with monoclinic lattice (JCPDS No 35-0134). Crystallites with a size of 10–20 nm were observed on dark-field images in the β-TCP (202) and HA (202) reflections (Figure 6c,f).

Thus, with the help of XRD and TEM methods it was found that the phase composition of both types of the coatings was similar. But the formation of the crystalline phases occurred as a result of different reactions. We assume that the following reactions took place during the formation of the coatings:

Ca_7.5_Sr_2.5_(PO_4_)_6_(OH)_2_ → Ca_(3-x)_Sr_x_(PO_4_)_2_ + Ca_(4-x)_Sr_x_(PO_4_)_2_O + H_2_Ogas (3)

(4) $$\beta -{\mathrm{Ca}}_{2}\mathrm{Sr}({\mathrm{PO}}_{4}{)}_{2}\;\overset{1125\;{}^{\circ}C}{\to }\;\alpha -{\mathrm{Ca}}_{2}\mathrm{Sr}({\mathrm{PO}}_{4}{)}_{2}$$

(5) $${\mathrm{Ca}}_{2}\mathrm{Sr}({\mathrm{PO}}_{4}{)}_{2}+H_{2}O\;\overset{\mathrm{pH}=10–11}{\to }\;{\mathrm{Ca}}_{\left(10-x\right)}{\mathrm{Sr}}_{x}({\mathrm{PO}}_{4}{)}_{6}(\mathrm{OH}{)}_{2}$$

3Mg^2+^ + 2PO_4_^3−^ → Mg_3_(PO_4_)_2_ (6)

HA is a thermally unstable phase. Monmaturapoj et al. [64] reported that dehydroxylation of HA to oxyapatite (Ca_10_(PO_4_)_6_O) occurs at a temperature of about 850–900 °C. Heating at temperature higher than 900 °C leads to the decomposition of HA into TCP and tetracalcium phosphate (TTCP) (reaction 3). TTCP is an unstable phase too and it transforms reversibly into HA.

The polymorphic transformation of β-TCP to α-TCP occurs at a temperature of 1125 °C (reaction 4) [42]. Moreover, as β-TCP and so α-TCP, which is more active in aqueous systems, may be involved in the formation of Ca-deficient hydroxyapatite (CDHA) [65]. Song et al. [66] also reported that when the coating containing β-TCP on AZ91D alloy was kept in NaOH solution, β-TCP turned into HA (reaction 5). Moreover, it is known that HA can be synthesized from aqueous solutions containing Ca^2+^ and PO_4_^3−^ in an alkaline medium at pH = 10–11 at high temperatures [65]. Thus, it was found that under the effect of high temperature micro-arc discharges TCP and secondary HA formed in Sr-HA coatings, whereas polymorphic transition and HA formation occurred in Sr-TCP coatings. Additionally, an interaction of electrolyte components with a magnesium substrate and the formation of magnesium phosphate Mg_3_(PO_4_)_2_ took place (reaction 6).

**Figure 7:**
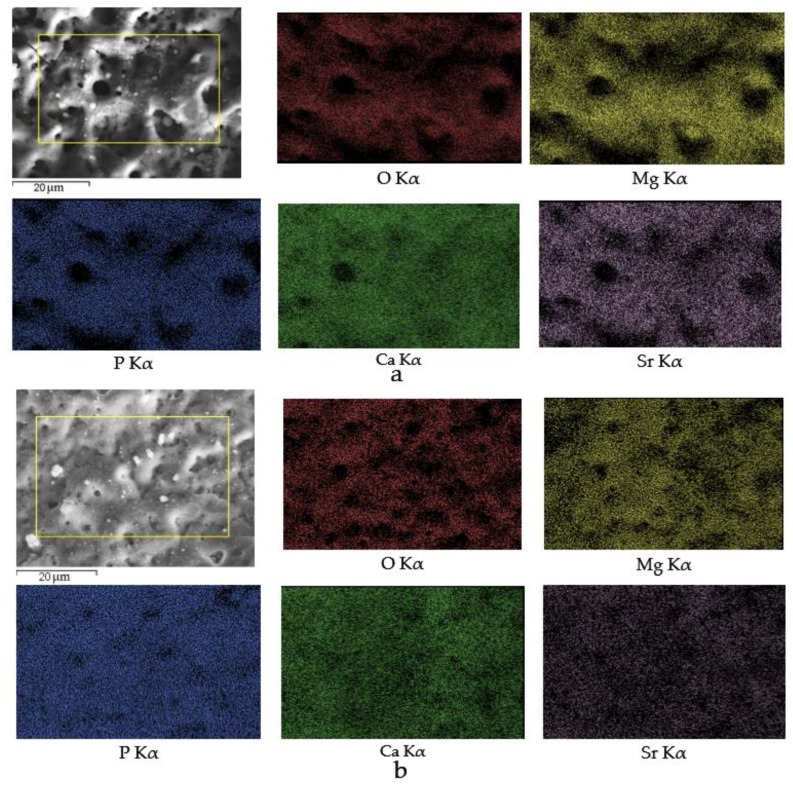
SEM image of the Sr-HA (**a**) and Sr-TCP (**b**) coatings deposited at 400 V; element distribution maps.

Elemental distribution maps (Figure 7) demonstrated that all elements including Sr were homogeneously dispersed in the Sr-HA and Sr-TCP coatings. This indicated that the Sr-substituted calcium phosphates HA and TCP have decomposed, and the ions Sr^2+^ released during the MAO process, under the influence of intense micro-arc discharge. The new amorphous and crystalline phases were formed with the participation of Sr.

The elemental composition of coatings deposited at different voltages of the MAO process is presented in Table 1.

Fluorine was not identified on the coatings surface, although NaF was introduced into the electrolyte to form a protective MgF_2_ layer. This thin inner layer present at the Mg/oxide interface and play a significant role in the corrosion resistance of the coatings [67].

The content of Sr was similar to both types of the coatings and was equaled to 1.8 ± 0.3 at.%. The Mg content was higher in Sr-HA than that in Sr-TCP. This is one more evidence that the MAO process proceeded more intensively in the presence of HA. In this case, the substrate material was actively involved in the process of coating formation during plasma chemical reactions. As the applied voltage increased, the quantity of calcium also increased while the magnesium content tended to decrease. This can be explained by the coating thickness growth and as a consequence increase of an amount of calcium phosphates deposited from electrolyte to the coatings.

The presence of Mg_3_(PO_4_)_2_ in Sr-HA and Sr-TCP was found by XRD and TEM. Moreover, the quantity of magnesium in the coatings exceeded the total content of calcium and strontium (Table 1). It should be assumed, that magnesium was included both in the amorphous phase and could replace calcium in the structure of the calcium phosphates.

**Figure 8:**
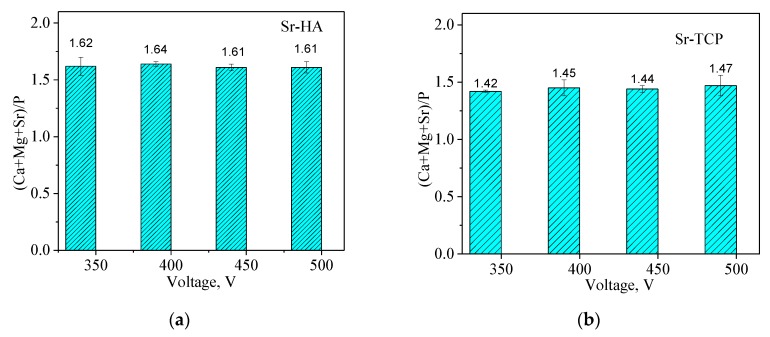
Diagrams of the (Ca + Mg + Sr)/P ratio against the applied voltage for the Sr-HA (**a**) and Sr-TCP (**b**) coatings.

To determine the ratio (Ca + Sr + Mg)/P and its change depending on the applied voltage (Figure 8), the elemental composition of the coatings was used. Sr-HA coatings were characterized by a higher (Ca + Sr + Mg)/P ratio than Sr-TCP coatings. The average value of the ratio (Ca + Sr + Mg)/P for Sr-HA and Sr-TCP coatings was 1.62 and 1.45, respectively, and was independent at the applied voltage (Table 1). The quantity of strontium in the coatings deposited at the voltages from 350 to 500 V varied in the range of 1.64–1.93 at.%.

### 3.3. Corrosion Behavior of the Sr-HA, Sr-TCP Coatings and Uncoated Mg0.8

**Figure 9:**
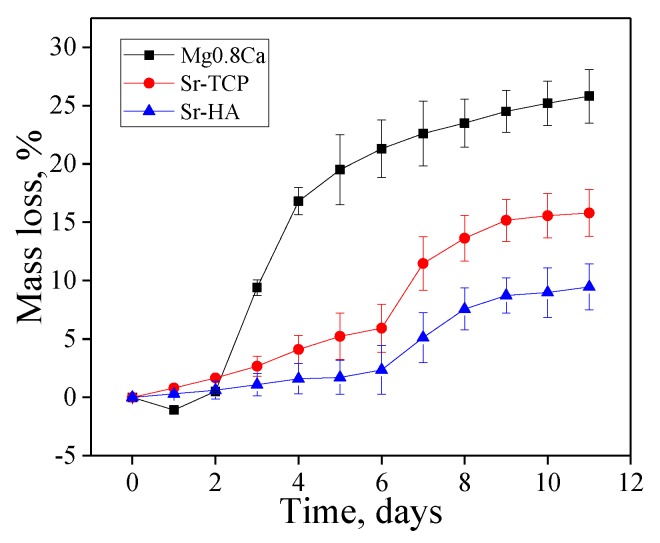
Graphs of the mass loss over time.

The study of the bioresorption process of bare magnesium alloy in 0.9% NaCl showed an increase in the mass of the samples by 1–2% during the first 2 days (Figure 9). Simultaneously with the dissolution and release of Mg^2+^ ions magnesium interacted with the solution. The reaction products formed and precipitated on the surface of the samples. The degradation mechanism of magnesium in aqueous solution can be presented as the following reactions [22]:

Mg + 2H_2_O → Mg^2+^ + 2OH^−^ + H_2_ (7)

Mg → Mg^2+^ + 2e^−^ (8)

2H_2_O + 2e^−^ → H_2_ + 2OH^−^ (9)

Mg^2+^ + 2OH^−^ → Mg(OH)_2_ (10)

The mass loss graph in the Figure 9 shows that the bioresorption rate of uncoated magnesium alloy was much higher compared to the coated magnesium samples. The both types of coatings performed a protective function and increased the corrosion resistance of the magnesium alloy. However, Sr-TCP coatings dissolved more intensely than Sr-HA ones. After 11 days of dissolution in 0.9% NaCl the loss of mass was 25%, 15%, and 8% for bare Mg0.8Ca, Sr-TCP, and Sr-HA, respectively.

**Figure 10:**
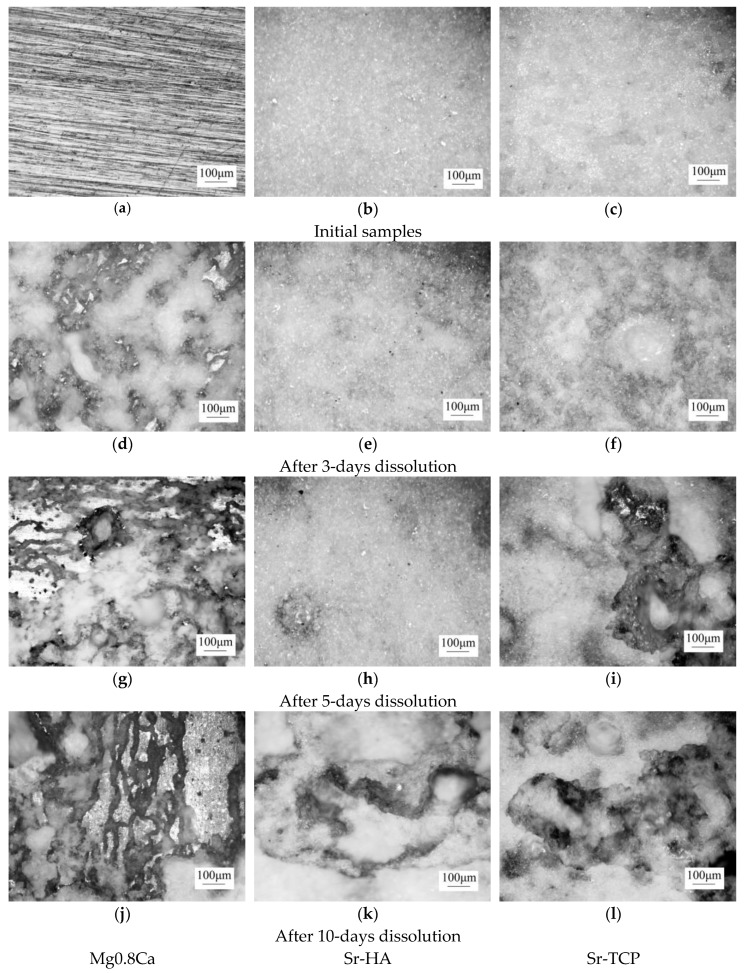
Optical images of the samples before the dissolution (**a**–**c**) and after 3 days (**d**–**f**), 5 days (**g**–**i**), and 10 days (**j**–**l**) of dissolution; (**a**,**d**,**g**,**i**)—bare alloy Mg0.8Ca, (**b**,**e**,**h**,**k**)—Sr-HA (500V) and (**c**,**f**,**i**,**l**)—Sr-TCP(500V) coatings.

The optical images in Figure 10 show that white gel-like products were formed on the surface of a dissolving magnesium plate. Even after 5 and 10 days of dissolution white sediment was presented on the surface of pure magnesium alloy, although the loss of mass was already 20–25% (Figure 10g,j).

The conducted analysis of optical images of the Sr-containing coatings revealed that Sr-TCP coatings dissolved more rapidly than Sr-HA ones. Dark areas and a rough relief formed on the surface of Sr-TCP coatings as a result of dissolution after 3 days (Figure 10f). After 5 and 10 days deep and extensive erosion zones were formed on the surface of Sr-TCP coatings (Figure 10i,l). For Sr-HA coatings the significant destruction was observed only after 10 days of immersion in 0.9% NaCl (Figure 10k).

### 3.4. Electrochemical Properties of the Sr-HA, Sr-TCP Coatings, and Uncoated Mg0.8Ca

**Figure 11:**
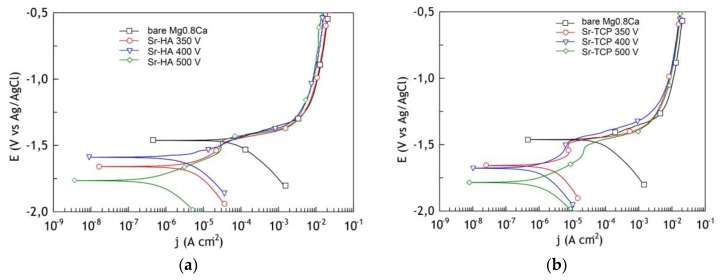
Potentiodynamic polarization (PDP) curves for bare Mg0.8 alloy, Sr-HA (**a**) and Sr-TCP (**b**) coatings recorded in 0.9% NaCl.

In the studied potential range, the curve of the uncoated sample had a form which is typical for magnesium [34]. The polarization curves for samples with Sr-HA and Sr-TCP coatings show substantial improvement of barrier properties compared to the curve for bare Mg alloy and exhibit substantial inhibition of both anodic and cathodic reactions. (Figure 11). The value of the corrosion current was almost ten times less for samples with coatings deposited at 500 V, compared with uncoated Mg0.8Ca.

The analysis of electrochemical parameters presented in Table 2 revealed that the coatings corrosion resistivity was almost ten times higher than that of a magnesium alloy. The Sr-HA coating obtained at the voltage of 500 V had the highest resistance value, impedance modulus, and the lowest value of the corrosion current. This can be explained as follows: first, at a voltage of 500 V, coatings with the maximum thickness were formed, and, second, HA with low solubility was mainly present in the phase composition of the Sr-HA coatings in accordance with the results of elemental analysis.

In our studies, the *E*_C_ values decreased with increasing of the formation voltage. However, in the case of coated magnesium alloy, being not uniform in chemical composition and has a complicated morphology, the higher values of *E*_C_ do not automatically indicate better barrier properties. In this case, a decrease in the corrosion potential of the coatings is explained by the superposition of potential-determining reactions of electrochemical dissolution of calcium salts and release of the Ca^2+^ ion during their interaction with a NaCl solution.

**Figure 12:**
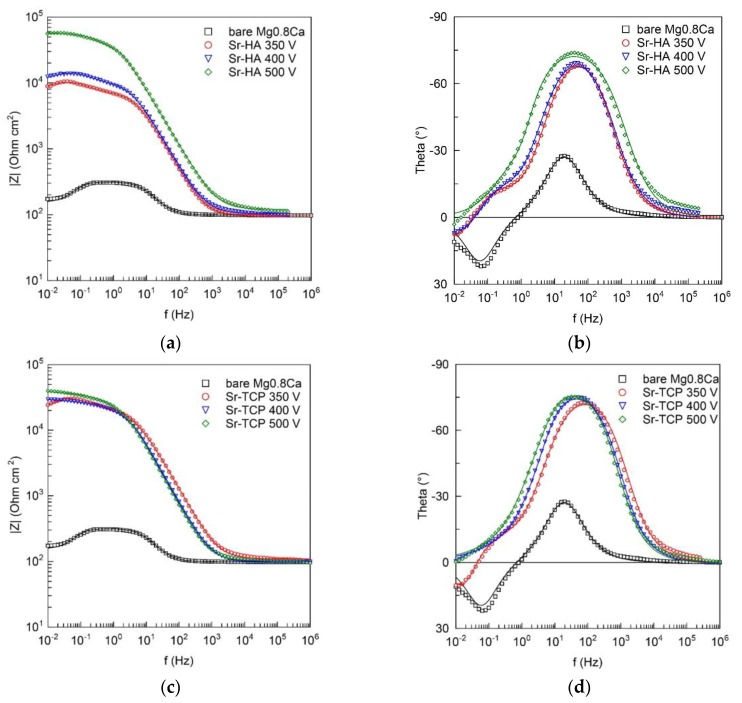
Bode plots, obtained in 0.9% NaCl for the bare Mg0.8Ca, Sr-HA (**a**,**b**) and Sr-TCP (**c**,**d**) coated samples.

Figure 12 presents the recorded Bode plots. The values of theta for the bare Mg0.8Ca at middle and low ranges indicate the presence of a thin film of natural oxide/hydroxide on the sample surface. The value of the impedance modulus |Z|_f→0 Hz_ in the low-frequency region was equal to 2 × 10^2^ Ω cm^2^ (Table 2), that means the material is very active and the protection is needed.

Heterogeneous layers, such as MAO-coatings, are usually modelled using CPE in equivalent electrical circuits (EECs) in order to simulate the electrochemical behavior of the samples.

Z_CPE_ = 1/[Q(jω)^n^] (11)

where ω—angular frequency, j—imaginary unit, and n and Q are the exponential coefficient and the frequency independent constant, respectively.

**Figure 13:**
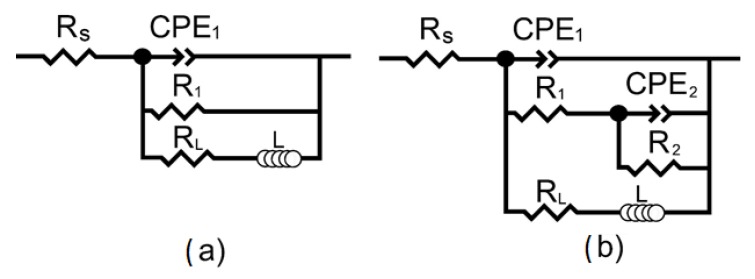
Equivalent electrical circuits (EECs) used for modelling the impedance spectra for: (**a**) bare Mg0.8Ca and (**b**) coated samples.

The values of the phase angle for both the bare Mg0.8Ca and coated samples showed the presence of both capacitive in the middle and inductive in the low frequency range loops. The R_1_–CPE_1_ capacitive loop for the bare sample is attributed to the charge transfer resistance and the capacitance of the electrical double layer (Figure 13a). For the coated samples, the R_1_–CPE_1_ circuit is ascribed to the electrolyte resistance in the pores and geometrical capacitance of the oxide layer, R_2_–CPE_2_ represent parameters of the barrier sublayer of the MAO-coating (Figure 13b). In both cases, R_L_–L chain is associated with the relaxation processes during the dissolution of magnesium followed by film formation, oxidation of magnesium at the corrosion front, hydrogen evolution on the filmed regions and with hydrogen evolution at the corrosion front [68]. Deposition of the corrosion products took place at the phase boundary of the alloy/electrolyte interface for untreated samples and the alloy/coating/electrolyte interface for the coated samples. The dissolution is preceded by adsorption of chlorides on the hydroxide layer [69]:

Mg(OH)_2_ + Cl^−^ → MgOHCl_ad_ + OH^−^ (12)

MgOHCl_ad_ + OH^−^ → Mg^2+^ + OH^−^ + Cl^−^ (13)

The inductance L values for all samples (Table 3) were far from the values, which are common in electrical engineering, which is associated with the formal use of this element, which is responsible in the EIS for slow adsorption and gas evolution.

Thus, it was found the Sr-HA and Sr-TCP coatings performed a protective function and reduced the corrosion rate of the Mg0.8Ca alloy. However, Sr-HA coating deposited at the 500 V demonstrated the highest corrosion resistance.

### 3.5. Biological Studies

**Figure 14:**
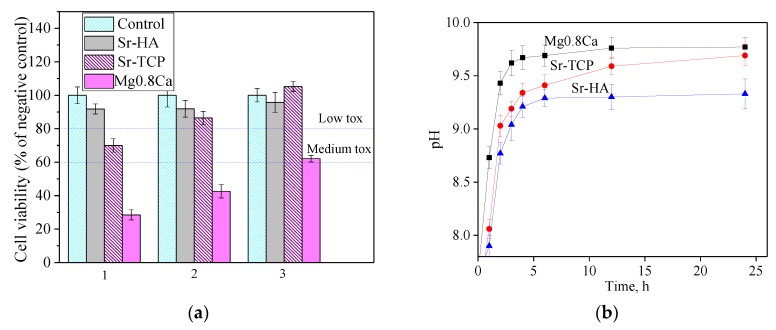
(**a**) Results of in vitro cytotoxic test of Mg.08Ca, Sr-HA and Sr-TCP samples extracts measured by an MTT assay (1); when extracts were diluted 10 (2) and 100 (3) times and (**b**) kinetics of pH changes in the cell medium during interaction with the samples.

The toxicity of the samples with respect to cell test cultures is the primary criterion for assessing the possibility of their use in biomedical applications. A comparative study of the toxicity of pure magnesium alloy and the samples with Sr-HA and Sr-TCP coatings using the MTT test allowed us to evaluate their effect on the viability of the L929 cell line (Figure 14a). The L929 cell line was provided by Vector (Koltsovo, Russia). The obtained data showed that Sr-HA coating did not significantly affect the cell line viability even without additional dilution of the extract. The samples with Sr-HA coating were non-toxic according to ISO 10993-5: 2009 (reduction in cell line viability did not exceed 20% relative to the negative control).

At the same time, the samples with Sr-TCP coating exerted a more pronounced effect on the mitochondrial cells activity. Cell viability was only 70% after the incubation with extracts of the Sr-TCP. In this case, the samples were characterized as medium toxic. When the extracts were diluted 10 and 100 times, cell viability increased to 86% and 105%, respectively. Thereby Sr-TCP samples became less toxic. For a bare Mg0.8Ca, cell viability was equal 28%. Thus, it was found that the Sr-HA and Sr-TCP coatings decreased the magnesium alloy toxicity, regardless of their composition. There was a significant difference in toxicity of uncoated magnesium alloy and modified coated samples.

One of the main problems of using magnesium alloy is its rapid dissolution in biological fluid, which leads to the formation of hydrogen and pH increase. In this research work a change in pH was observed when the samples were placed in cell culture medium (Figure 14b). The hydrogen evolution during the dissolution of the uncoated Mg0.8Ca (reaction 5) and samples with coatings for the day of exposure led to an increase in the pH value of the cell medium to 9.8. The lowest pH values were demonstrated by samples with the Sr-HA coating. The obtained results correlated well with toxicity data.

## 4. Discussion

Comparative studies of the formation, structure, and properties of Sr-HA and Sr-TCP coatings on Mg0.8Ca substrates were carried out and interesting results were obtained. The coatings had a gradient porous structure. The particles of calcium phosphates were uniformly distributed on the surface of the coatings. This structure can be explained by the mechanism of coatings formation by MAO method. Due to anode potentiostatic mode, the discharges with maximum intensity were realized at the initial moment of the deposition process. The current density at this moment was maximum and reached of 0.35 A/cm^2^. The components of the electrolyte decomposed in the strong discharges and porous coating were formed as a result of plasma chemical reactions. Hussein et al. [63] described such a mechanism of the pore structure formation when coatings deposited in a unipolar current mode. Within 3–4 min the intensity of micro-arc discharges decreased, and the process was completed (Figure 1a). The particles of Sr-substituted calcium phosphates deposited on the coatings surface at this moment. However, the residual discharges affected the particles so a partial change in the structure of calcium phosphates have taken place. Similar mechanism of micro-arc coating formation was confirmed in other previous researches [33,34].

It was found that the MAO process was more intensive in the electrolyte containing HA. Higher values of the thickness and surface roughness R_a_ of Sr-HA coatings indicated this fact. Pores uniformly distributed over the surface of both types of the coatings were two-times larger in Sr-HA, which means that stronger micro-arc discharges took place in this case. HA is less resistant to high temperatures than TCP. Monmaturapoj et al. [64] reported that decomposition of HA occurred at the temperatures above 900 °C in the plasma of a micro-arc discharge. In addition, it is known that [42] polymorphic transition β-TCP → α-TCP took place at 1125 °C. Moreover, the α-TCP modification is stable up to 1400 °C. Consequently, a larger amount of ions was involved in the process at the same voltages in the electrolyte with HA. This assumption was confirmed by a higher quantity of the amorphous phase contained in Sr-HA coatings.

The authors [38] also note that when the molten substance was cooled in the channel of a fading micro-arc discharge, new compounds were formed in addition to the amorphous phase. Polymorphic transition β-TCP → α-TCP and the secondary HA formation occurred in both types of the coatings. Magnesium phosphate was also formed as a result of the interaction of a magnesium substrate with electrolyte components. Darband et al. [36] also confirm this phenomenon. According to the elemental composition, a large amount of Mg was found in the coatings in addition to Ca and Sr. We supposed that magnesium was involved not only in the magnesium phosphate and in the amorphous phase, but also replaced Ca in the structure of calcium phosphates together with strontium. The ratio (Ca + Sr + Mg)/P was equal 1.64 and 1.47 for Sr-HA and Sr-TCP coatings, respectively. Thus, it can be assumed that the composition of Sr-HA and Sr-TCP coatings was predominantly presented by (Sr,Mg)-substituted hydroxyapatite and (Sr,Mg)-substituted tricalcium phosphate. The behavior of the coatings during the bioresorption in a model biological fluid confirmed this assumption. The solubility of Sr-HA was lower than that of Sr-TCP because hydroxyapatite is known to be more resistant to dissolution than tricalcium phosphate [43,65]. The studies of the coatings electrochemical properties also showed that corrosion resistance of Sr-HA coatings deposited at 500 V was higher than that of the Sr-TCP coatings.

As a result of biological studies, it was revealed that the coating reduced toxicity of Mg0.8Ca alloy regardless of the coatings composition. There was a significant difference in the toxicity of the uncoated magnesium alloy (28% of living cells) and samples modified by coatings. Moreover, cell viability after incubation with extracts of Sr-HA was higher than that for Sr-TCP. Sadowska et al. [70] showed the different effect of calcium phosphates on the processes of osseointegration. β-TCP reduced the release of inflammatory cytokines more intensively than calcium deficient hydroxyapatite (CDHA). However, after macrophage cultivation, CDHA showed stronger osteogenic effects, contributing to the osteogenic differentiation of BMSC and SaOS-2 cells. Further comparative studies of the osteogenic properties of Sr-HA and Sr-TCP coatings are required.

## 5. Conclusions

The coatings containing Sr-HA and Sr-TCP were formed on the Mg0.8Ca substrates by the MAO method in the range of the process voltages of 350–500 V. Sr-HA coatings were characterized by the higher values of thickness, roughness R_a_, and pore sizes than Sr-TCP. The coatings had a similar phase composition and, in addition to the amorphous phase, contained crystalline phases such as β-TCP, α-TCP, Mg_3_(PO_4_)_2_, and hydroxyapatite. Both types of coatings have demonstrated protective properties and high corrosion resistance. However, the Sr-HA coating was less soluble and had greater corrosion resistance than Sr-TCP. During Sr-HA dissolution, the pH of the medium slightly increased and cell survival was higher than for Sr-TCP coatings. However, after the dilution of the Sr-TCP extract, their cytotoxicity became lower.

## Figures and Tables

**Table 1:** The elemental composition of the Sr-HA and Sr-TCP coatings on Mg0.8Ca determined by the EDX method in the region, (at.%).

Elements	Sr-HA				Sr-TCP			
	350 V	400 V	450 V	500 V	350 V	400 V	450 V	500 V
O	64.6	66.5	68.0	68.2	64.0	64.4	64.0	66.7
Mg	13.2	10.3	9.0	8.3	10.5	8.4	7.2	6.1
Ca	7.0	8.6	8.8	9.0	8.9	10.9	12.1	11.8
Sr	1.7	1.9	1.9	1.9	1.6	1.8	1.9	1.9
P	13.5	12.7	12.3	11.9	14.8	14.5	14.7	13.4

**Table 2:** Calculated electrochemical parameters of the samples.

Sample	*E*_C_, Corrosion Potential V (Ag/AgCl)	*j*_C_, Corrosion Current A cm^−2^	*R*_p_, Corrosion Resistance Ω cm^2^	|Z|_f→0 Hz_, Impedance Modulus Ω cm^2^
bare Mg0.8Ca	−1.35	7.0 × 10^−6^	4.3 × 10^3^	2.0 × 10^2^
Sr-TCP 350 V	−1.66	2.5 × 10^−6^	1.4 × 10^4^	2.4 × 10^4^
Sr-TCP 400 V	−1.68	1.5 × 10^−6^	2.8 × 10^4^	2.9 × 10^4^
Sr-TCP 500 V	−1.78	8.9 × 10^−7^	3.9 × 10^4^	3.9 × 10^4^
Sr-HA 350 V	−1.66	8.3 × 10^−6^	7.6 × 10^3^	8.9 × 10^3^
Sr-HA 400 V	−1.59	2.5 × 10^−6^	1.2 × 10^4^	1.2 × 10^4^
Sr-HA 500 V	−1.77	5.8 × 10^−7^	6.4 × 10^4^	5.6 × 10^4^

**Table 3:** Calculated parameters of the EECs elements *.

Sample	CPE_1_		R_1_ (Ω cm^2^)	CPE_2_		R_2_ (Ω cm^2^)	R_L_ (Ω cm^2^)	L (H cm^2^)
	Q_1_ (S cm^−2^ s^n^)	n		Q_2_ (S cm^−2^ s^n^)	n			
Mg0.8Ca	1.1 × 10^−4^	0.91	217.1	–	–	–	78.0	555.2
Sr-TCP 350 V	2.6 × 10^−6^	0.88	2.0 × 10^4^	9.0 × 10^−5^	0.46	3.3 × 10^4^	4.2 × 10^4^	4.0 × 10^5^
Sr-TCP 400 V	3.8 × 10^−6^	0.90	2.4 × 10^4^	2.3 × 10^−5^	0.44	3.6 × 10^4^	6.5 × 10^4^	1133
Sr-TCP 500 V	4.0 × 10^−6^	0.91	2.4 × 10^4^	1.5 × 10^−5^	0.42	8.2 × 10^4^	6.8 × 10^4^	1190
Sr-HA 350 V	6.8 × 10^−6^	0.89	7.8 × 10^3^	3.0 × 10^−4^	0.83	3.9 × 10^3^	2.6 × 10^4^	3.7 × 10^5^
Sr-HA 400 V	6.7 × 10^−6^	0.88	1.0 × 10^4^	2.1 × 10^−4^	0.84	5.0 × 10^3^	4.4 × 10^4^	4.3 × 10^5^
Sr-HA 500 V	3.5 × 10^−6^	0.86	5.4 × 10^4^	1.5 × 10^−5^	0.86	4.6 × 10^4^	1.4 × 10^5^	1.4 × 10^3^

* The fitting curves (solid lines in Figure 12) were ploted with help of these parameters.
